# Confrontation of the increase in ELWI rate regarding the septic polytraumatised patient administering furosemide: is it effective?

**DOI:** 10.1186/cc10851

**Published:** 2012-03-20

**Authors:** P Sarafidou, E Pappa, D Dimitriadou, D Litis, I Pavlou

**Affiliations:** 1 KAT General Hospital Kifisia, Athens, Greece

## Introduction

The ELWI rate (measurable with the PICCO catheter) conveys the extravascular lung water. The increase of the ELWI rate is a frequent finding in heavily septic patients and it is connected with very high mortality. The purpose of this study is to find out if the administration of furosemide is possible to reduce effectively the ELWI rate and if this decrease can be maintained regardless of the confrontation of the sepsis.

## Methods

We studied 20 septic poytraumatized patients (mean ISS score 35) with ARDS syndrome and ELWI >10, with good renal function and on medical treatment with levophed. We administered furosemide 10 mg/hour, for 24 hours, while at the same time we confronted the septic source. During all these we noted PO_2_/FiO_2_, MAP, CVP and ELWI every 8 hours. Moreover, we noted the changes in the levophed dose and the total balance of fluid at the end of the 24-hour interval.

## Results

The ELWI rate was up 12 to 16 before the administration of furosemide. We marked that after the administration of furosemide and at the end of the first 8-hour interval, the ELWI rate decreased about 2 to 3 units but we had to increase the dose of vasoconstriction, until the end of the 24-hour interval the ELWI rate restored to the initial high level and could not manage to decrease the dose of vasoconstriction to have a negative balance of fluids. The improvement of PO_2_/FiO_2 _was insignificant statistically and we confronted operatively the septic source for five to 20 patients at the end of the 24-hour interval.

## Conclusion

We managed to decrease very little the ELWI rate, only temporarily after the administration of furosamide about a 24-hour interval. The small improvement of the PO2/FiO2 finally leads to a decrease of the ELWI rate but it is not important statistically and on the other side leads to an increase of vasoconstriction. Therefore, this method is not effective. We gain only a short time for the safer surgical treatment if it is needed.

